# Towards a greater engagement of universities in addressing climate change challenges

**DOI:** 10.1038/s41598-023-45866-x

**Published:** 2023-11-03

**Authors:** Walter Leal Filho, Sebastian Weissenberger, Johannes M. Luetz, Javier Sierra, Izabela Simon Rampasso, Ayyoob Sharifi, Rosley Anholon, Joao Henrique Paulinho Pires Eustachio, Marina Kovaleva

**Affiliations:** 1 Department of Natural Sciences, Manchester Metropolitan University, Chester Street, Manchester, M1 5GD UK; 2 European School of Sustainability Science and Research, Hamburg University of Applied Sciences, Hamburg, Germany; 3 TÉLUQ University, Quebec City, Canada; 4 Graduate Research School, Alphacrucis University College, Brisbane, QLD Australia; 5 School of Law and Society, The University of the Sunshine Coast, Maroochydore, QLD Australia; 6 School of Social Sciences, The University of New South Wales, Sydney, NSW Australia; 7 Department of Applied Economics, Faculty of Law, Research Center On Global Governance, Educational Research Institute, University of Salamanca, Paseo Tomas y Valiente, Salamanca, Spain; 8 Departamento de Ingeniería Industrial, Universidad Católica del Norte, Antofagasta, Chile; 9 The IDEC Institute & Network for Education and Research on Peace and Sustainability (NERPS), Hiroshima University, 1-5-1 Kagamiyama, Higashi Hiroshima City, Hiroshima, Japan 739-8529; 10 School of Architecture and Design, Lebanese American University, Beirut, Lebanon; 11 School of Mechanical Engineering, University of Campinas, Campinas, Brazil

**Keywords:** Climate change, Environmental impact

## Abstract

Many higher education institutions around the world are engaged in efforts to tackle climate change. This takes place by not only reducing their own carbon footprint but also by educating future leaders and contributing valuable research and expertise to the global effort to combat climate change. However, there is a need for studies that identify the nature of their engagement on the topic, and the extent to which they are contributing towards addressing the many problems associated with climate change. Against this background, this paper describes a study that consisted of a review of the literature and the use of case studies, which outline the importance of university engagement in climate change and describe its main features. The study identified the fact that even though climate change is a matter of great relevance to universities, its coverage in university programmes is not as wide as one could expect. Based on the findings, the paper also lists the challenges associated with the inclusion of climate change in university programmes. Finally, it describes some of the measures which may be deployed in order to maximise the contribution of higher education towards handling the challenges associated with a changing climate.

## Introduction

Many universities worldwide are continuously showing their commitment to preparing students for a role in society where they can contribute to climate change mitigation and adaptation^1^. Education plays an important role in changing people's behaviour and attitudes; young people in the classrooms can learn about the impacts of climate change and how to mitigate and adapt to it, and they can be motivated to act^2^. The university’s role in relation to climate change education is critical in addressing scientific, environmental, social, and political challenges. Future decision-makers need to make their decisions from an informed position, and for this reason, climate change education and research programmes are of major importance^3^. Higher education institutions (HEIs) are part of both the solution and the problem regarding climate change^4^. By becoming actively engaged in efforts against climate change, HEIs can provide research-based and educational solutions to identify the most critical climate impacts and ways to handle them. Institutions can operate as hubs by creating, testing, and disseminating information about climate mitigation and adaptation strategies. Furthermore, HEI often undertakes research activities and seize upon opportunities to generate innovative knowledge that can help their local communities to adapt to climate change^5^. They deliver significant engagement and provide a platform for designing, testing and implementing innovative practices which may help in efforts to address the many impacts of climate change, locally, nationally, and globally. For instance, universities are among the key players in exploring and developing effective carbon pricing solutions including their economic feasibility and stimulating investments to reduce the technologies’ costs^6^. In the light of additional pressure posed by climate change on healthcare systems worldwide, it is essential to strengthen educational and training programs by introducing ‘climate change’ into medical school curricula and students’ activities. This will ensure that graduate health professionals acquire knowledge and skills to diagnose and respond to the health threats and impacts of climate change and understand public health issues^7,8^. Another role universities play in affecting climate action-related transformational change is through their engagement in advocacy and activism^9^. For instance, in the United States and Canada HEI have been involved in the fossil fuel divestment (FFD)^10,11^. In the United States, campaigns, primarily led by students, focus on justice including social, environmental, and economic issues^10^. In Canada, the campaigns use the signing of sit-ins, petitions, protests and rallies as well as branding and messaging from international environmental organizations^11^.

On the other hand, universities are contributors to climate change and hence, often feel an obligation to address individual impacts by greening their campuses. Many HEIs around the world have adopted initiatives such as the ‘carbon neutral university’ converting to low-emission or carbon–neutral organisations. As examples of these initiatives, the University for Sustainable Development in Eberswalde and Leuphana University both in Germany, are on a path to becoming carbon–neutral^12^. Others are engaging in initiatives to handle climate change as part of their efforts in the field of sustainable development^13^. In addition to carbon neutrality and waste management, universities aim to improve materials and resource use efficiency, environmental quality, retrofitting residential and non-residential construction buildings, and increase green areas and use of green transportation. For instance, Arizona State University, one of the largest public universities in the USA, with almost 100,000 students and employees, reported the achievement of carbon neutrality in 2019^14^. Development of green campuses in China focuses on energy and resource efficiency through introducing energy-saving technology in campus buildings and facilities, energy statistics and auditing, as well as energy-saving operations. All these initiatives are strongly supported by the national government through policies and financial tools^15^. In Italy, the largest campus in the country of the University of Calabria (UNICAL) has significantly improved its energy systems through the use of photovoltaic, solar, and geothermal energy produced on campus^16^. Additionally, by introducing internal carbon pricing, universities could demonstrate practical implications for emission reduction through waste management^17^ and energy use^18^. Climate change education and approaches to greening campuses are also considered among the university's strategies to contribute to sustainable development^19^. The strong linkage between these fields contributes to overcoming challenges in attaining the goals of the other. Sustainable Development Goals (SDG) 13, particularly Target 13.3, aims at “improving education, awareness-raising and human and institutional capacity on climate change mitigation, adaptation, impact reduction, and early warning”. Furthermore, the wide range of initiatives launched to foster climate change literacy and education including the UNESCO Climate Change Education for Sustainable Development Programme^20^. The program contributed to advancing such topics as sustainable development and climate change in national curricula and educational standards across the countries^21^.

Most of the current studies report on one or several aspects concerning HEIs efforts to tackle climate change, like the aforementioned examples. Therefore, there is a need for studies that identify the overall nature of HEIs participation, and the extent to which they are contributing towards addressing the many problems associated with climate change. This paper explores universities' engagement in addressing the threats posed by climate change, its main features, potential measures towards its maximisation, and associated challenges worldwide. To achieve this goal, this research consisted of a review of the literature and the use of case studies, which outline the importance of university engagement in climate change and describe its main features. The consequent sections describe methods used, obtained results, and lessons learned. The paper concludes by summarising the main findings and describing measures that higher education institutions should deploy in the long term, to better address climate change.

## Methods

The objective of this study is to find out what climate change-related themes and topics have been pursued by universities. One way to answer this question is to examine publications that have focused on issues related to climate change education and research programs and initiatives in academic institutions. For this purpose, we relied on bibliometric analysis techniques as they can highlight key terms that have been used in the literature and their interactions. Various software tools such as CiteSpace, SciMAT, and VOSviewer are available for bibliometric analysis. Here, we used the latter as its term co-occurrence maps are more detailed and easier to interpret^22,23^. The input data for bibliometric analysis can be obtained from academic literature databases such as Scopus and the Web of Science. In this study, we used the Web of Science for its reputation to index quality peer-reviewed literature. To retrieve relevant literature for inclusion in the analysis, we developed a search string that is a combination of terms related to climate change, impacts of climate change, teaching and research programs, and academic institutions.

The full search string is available in the “Appendix”. It was created to embrace the main topics related to this research (inclusion criteria), with a structure of four main blocks. The first is related to terms related to climate change and encompasses variations commonly used in the literature such as ‘global warming’, ‘climate variability’, etc. The second block of terms is related to ‘extreme events’, while the third brings some practices of universities such as education, teaching, training, curricula, research, etc. Finally, the last section of the search string was created due to the focus selected in this study, which is to understand the perspective of higher education institutions. It is worth considering, however, that the terms chosen might now encompass the totality of possible terms related to climate change since there is a huge variety used throughout the literature. The authors are aware of this issue and brought this discussion as a limitation in the conclusions section.

**Figure 1:**
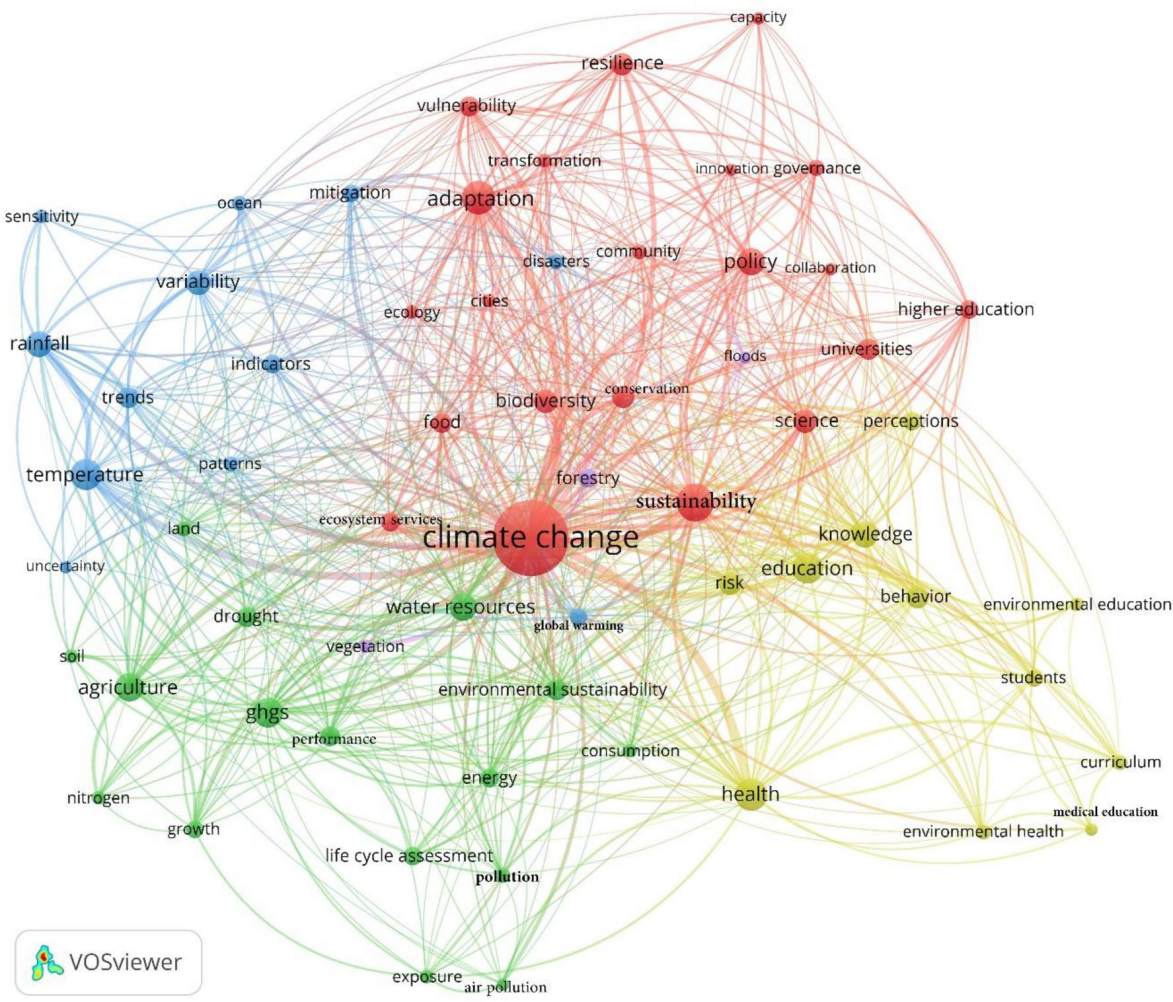
Results of the term co-occurrence analysis.

The initial literature search was conducted on July 18, 2022, and returned 1214 documents. These documents were screened to only include those that show how climate change education and/or research is pursued by universities (exclusion criteria). At the end of the screening process. A total of 794 documents remained in the database and were used for term co-occurrence analysis in VOSviewer. The co-occurrence analysis was done in several steps to ensure obtaining the most accurate outputs. To be more specific, after the initial analysis, we found out many synonyms need to be merged (e.g., ‘climate change’ and ‘climate-change’). For this purpose, we developed a thesaurus file and added it to the software. The process was repeated until no synonyms were found in the output. The final output (Fig. 1) is a network of nodes and links, where node size is proportional to the occurrence to frequency (of terms) and link width is proportional to the strength of connections between terms. Closely connected terms form clusters that can be interpreted as major thematic areas that have received relatively more attention in the literature. In this perspective, it was possible to label clusters manually since the number of clusters formed and the terms extracted were manageable. To label the clusters, the authors analysed the relationship of terms of a specific cluster and provided a label representing the discussion embedded in each one of the clusters^24^. These will be further explained in the results section.

In addition to this, we completed the literature review by selecting a set of key case studies regarding four core areas for university engagement, namely (1) research and development, (2) teaching and learning, (3) governance and operations, and (4) civic engagement and community outreach. We performed a literature mapping using the Web of Science database to identify the case studies. This was completed using a general search using Google and recognised case studies implemented by universities in different countries. Relevant case studies were selected by the research team using the following criteria: number of citations, degree of innovation, diversity of knowledge and research areas, geographical diversity, and potential for replication and mainstreaming in other contexts. Four tables were designed with selected examples, which entail a specific set of information, namely the type of climate change work undertaken, the main purpose of the implemented initiative, the name of the participating universities, and the country. Also, to ensure the tracing of the information, the tables contain bibliographical references or web links. This also allows a cross-check of the information and enables readers to obtain further details.

Using this literature mapping approach identified, collected, and analysed the existing literature on specific case studies of interest. This process made it possible to highlight and synthesise key issues from the selected literature, thus providing clear insights into the state of knowledge on key case study topics. Accordingly, literature mapping conceptualised a range of possible future research directions, policy implications, and/or practical recommendations for different stakeholder groups. Crucially, surveying the state of the art in this manner helped the researchers to avoid duplicating previous work, identify areas where further investigation is needed, and enable the development of strategies for evidence-based decision-making that researchers, policymakers, and practitioners may leverage in different contexts.

## Results and discussion

### Bibliometric assessment

Figure 1 shows that multiple topics related to climate change adaptation and mitigation have been addressed in the context of higher education. This is evidenced by the diversity of terms in each of the four clusters which can be considered as research strands explored by the literature in the field. The output of the term co-occurrence analysis (Fig. 1) shows that multiple topics related to climate change adaptation and mitigation have been addressed in publications on climate change-related education and/or research activities/programs.

The red cluster describes aspects related to general policies aimed at reducing vulnerabilities and enhancing resilience and adaptive capacity, embracing terms related to biodiversity, food systems, and ecosystem services. Studies in this cluster usually discuss the relevance of governance in HEIs to ensure universities’ contribution towards reducing their impact, implementing adaptation strategies on climate change vulnerabilities, and fostering sustainable development^25–27^. This governance perspective is relevant since it could contribute to the universities’ process of ensuring that desired practices are initiated, implemented, and continued by the several stakeholders engaged in the process^28^. This perspective also implies the adaptation policies that aim to assist the universities in assembling their several systems, tackling the university’s campus operations, and helping society by producing research on the climate change field, especially related to themes such as ecology, food, and biodiversity. Ecology and biodiversity policies in the context of higher institutions are also discussed, especially in the context of green spaces, generating externalities in the perspective of ecological function and urban communities^29^ and fostering the discussion of how to maintain biodiversity in a context of climate change adaptation since many species are constrained by changes in climate^30,31^. This cluster also presents studies on campus as ecosystems, exploring whether humans and the biosphere could be reconnected, enhancing the awareness of how to deal with the biodiversity loss related to climate change^32^.

The yellow cluster is focused on climate and environmental education and risk reduction. This cluster, in particular, focuses on the educational practices of universities and the extent to which they address climate change and environmental challenges^33–36^. More specifically, it deals with the knowledge and sustainability behaviour of students in the process of educating well-versed agents in climate change aspects and capable of conducting adaptation and mitigation strategies^37,38^. This cluster also reports on integrating disaster reduction for extreme events^39–41^, highlighting the significance of disaster risk education and environmental awareness programs to effectively address these challenges^42^.

Studies that belong to the blue cluster, in turn, are mainly focused on assessing the temperature, precipitation, and other aspects of climate change variability^43–45^. The relation this cluster has with HEIs is mainly focused on research practices, where research centres contribute to assessing climate change challenges by finding patterns and estimating indicators related, for example, to rainfall, temperature, and extreme events^46,47^. For example, Stefanidis and Alexandridis^48^ studied the temporal variability, precipitation trends, and evapotranspiration in two forest regions in Greece. They discussed the drought scenarios and the implications for climate change adaptation. Similarly, Rawat and colleagues^49^ analysed the rainfall variability and intensity of long-term monthly rainfall data using the Precipitation Concentration Index, which, according to the authors, could prepare governments for extreme weather events, which are imperative to adaptation to climate change conditions.

Finally, the green cluster is the second in a number of terms and has two main discussion streams. The first one is related to the climate change impact on water resources, land, and soil degradation, and inducing droughts^50–55^. The second perspective this cluster highlights is related to the precedents of climate change as well as the adaptation and mitigation strategies to address the challenges related to climate change. For example, there are reports on the potential of organic agricultural systems instead of crop productions using nitrogen-based fertilisers, since it leads to reduced N_2_O emissions^56–60^, the importance of renewable energy production systems^60,61^ as well as the industrial and human activities which can contribute to the emission of GHGs and environmental pollution^62^, impacting negatively human health^63,64^.

## Cases

### Research and development

Climate change research has been led since 1896 with Svante Arrhenius founding paper^65^. Since then, the volume of scientific literature on climate change has been increasing rapidly. The total number of articles on climate change exceeds 120 000 up to 2015^66^; almost 90 000 papers were published between 1991 and 2011^67^. New fields of research have merged over time, often responding to society’s needs, such as attribution science, first documented in 2004^68^ and included in the IPCC AR5^69^, the study of social health impacts of climate change, the incorporation of traditional ecological knowledge and indigenous perspectives, and generally speaking a growing emphasis on adaptation, including novel approaches like community-based adaptation^70^ and participatory action-research^71–74^. Table 1 presents a set of elected case studies on research institutions.

**Table 1:** Case studies of research institutions.

Institutions	Purpose	University	Country	Website
Prairie Climate Centre	Mapping, documenting, recording, researching, and communicating climate change	University of Winnipeg	Canada	https://prairieclimatecentre.ca/
Réseau Inondations InterSectoriel du Québec (RIISQ)	Development of advanced transdisciplinary research on flood risk management and its consequences	All universities in Quebec	Canada	https://riisq.ca/en/home/
Ouranos consortium on Regional Climatology and Adaptation to Climate Change	Innovation cluster and consultation forum enabling better adaptation to climate change, combines regional climate modelling and other approaches from natural and social sciences	NGO supported by UQAM, McGill, Laval, and INRS universities + other partners	Canada	https://www.ouranos.ca/en/
Potsdam Institute for Climate Impact Research	Inter-disciplinary climate impact research for global sustainability and contributing knowledge and solutions for a safe and just climate future	NGO member of the Leibnitz Association, researchers affiliated with the University of Potsdam, Humboldt University Berlin and others	Germany	https://www.pik-potsdam.de/
Stockholm Resilience Center	Understanding the complex dynamics of people and planet in the Anthropocene	Stockholm University	Sweden	https://www.stockholmresilience.org/
Munasinghe Institute for Development (MIND)	Sustainomics is defined as "a transdisciplinary, integrative, comprehensive, balanced, heuristic and practical framework for making development more sustainable	Independent organisation that works with universities in Brazil, China, Germany, India, South Africa, Sweden, the UK, USA	Sri Lanka	http://www.mindlanka.com
International Centre for Climate Change and Development (ICCCAD)	Climate change adaptation and development closely related to local experience, knowledge, and research	Partnership between the Independent University, Bangladesh (IUB), Bangladesh Centre for Advanced Studies (BCAS) and the International Institute for Environment and Development (IIED)	Bangladesh	https://www.icccad.net/

At the same time, climate change research has become more interdisciplinary and transitioned from individual researchers to research centres, hosted by one or several institutions. It also more often than before involves stakeholders from society, leading to collaborative research initiatives. We illustrate this through examples for all three types of research centres in Canada: single-university—the Prairie Climate Centre, multi-university—the Réseau Inondations InterSectoriel du Québec (RIISQ), collaborative, and Ouranos. Two of the most influential research centers, both in numbers and impact of publications, are the Stockholm Resilience Centre and the Potsdam Institute for Climate Impact Research, which pioneered ground-breaking work on planetary boundaries, climate tipping points, and exploration of past and future climates in an interdisciplinary perspective^75–78^. It must be stressed that climate change research is not an exclusivity of European or North American universities. Institutions like the Munasinghe Institute for Development and the International Centre for Climate Change and Development are highly respected in the fields of adaptation or sustainability applied to climate change and incorporate issues, approaches, and values relevant to the Global South in their research. As we wish to demonstrate through the selected research projects below, there is a trend for the development of international, interdisciplinary cross-institution initiatives in climate change research, certainly also favoured by funding agency policies, especially in the research and development sector. Such projects can have a real impact on the ground; however, they need careful scientific and organisational planning in order to be truly successful^79^. Table 2 presents a set of elected case studies on research projects.

**Table 2:** Case studies of research projects.

Project	Purpose	University	Country	Website
The Inter-University Sustainable Development Research Programme	Promotion of climate change and sustainability research around the world	Manchester Metropolitan University and numerous partner institutions on all 6 continents	UK	https://www.mmu.ac.uk/environmental-science-research/inter-university-sustainable-development-research-programme
"Adaptation Research a Trans-disciplinary Transnational Community and Policy Centred Approach" (ARTisticc)	Apply innovative standardized transdisciplinary approaches to develop robust, socially, culturally and scientifically, community-centred adaptation strategies as well as a series of associated policy briefs, translation into stories and artwork	Université de Versailles Saint-Quentin-en-Yvelines, Institut Universitaire Europeen de la Mer, Cochin University of Science and Technology, North-Eastern Federal University, Université Cheikh Anta Diop, Université de Moncton, University of Alaska	Canada, France, India, Russia, Senegal, USA	http://artisticc.net/
Deltas, vulnerability and Climate Change: Migration and Adaptation (DECCMA)	Evaluatioin of the effectiveness of adaptation options in deltas in Africa and Asia	Institute of Water and Flood Management, University of Engineering and Technology, Jadavpur University, University of Ghana, University of Southampton	Bangladesh, Ghana, India, UK	http://generic.wordpress.soton.ac.uk/deccma/
Co-construction of adaptation scenarios for coastal zones in the context of climate change	Adaptation to coastal risk scenario building based on physical and social vulnerability indicators in collaboration with decision-makers, including storylines and serious games	Institut Universitaire Européen de la Mer, Université du Québec à Rimouski and 4 other universities	France, Canada	https://arico.uqar.ca/

### Teaching and learning

Equipping graduates with the necessary skills and capabilities required to succeed in both their personal and professional lives is a crucial goal for higher education institutions. In higher education institutions, there are varied interpretations of the cultural, social, economic, and environmental aspects of sustainable development. Simultaneously, teachers do not reach a consensus on how these different dimensions are interconnected^80^. Furthermore, there are diverse perspectives on how these matters should be approached within various degree programs and courses, and this can influence students' perceptions of values related to sustainability, ethics, and social responsibility^81^. However, understanding the complexity of climate change may be challenging for students and educators^82^. This could clarify why students' awareness of sustainability issues is not uniform or consistent^83^. However, it also underscores the potential intricacy associated with enhancing awareness of social, economic, and environmental issues among students^84^. From this viewpoint, students should acquire the skills not just to translate innovative ideas into tangible projects but also to effectively integrate environmental, social, and financial goals^85^.

In this regard, it is vital to increase their interest in the UN Sustainable Development Goals (SDGs), as well as prepare graduates to implement real-life solutions based on sustainability criteria^86^. Against this background, it is necessary not only to identify misconceptions and guarantee a proper understanding of climate change’s roots and consequences but also to help students become active and critical citizens capable of facilitating real change. In this context, the integration of SDGs in higher education requires the identification and clarification of educational objectives, as well as the adoption of innovative teaching and learning strategies suitable to transform education^87^.

Table 3 presents a set of selected case studies on teaching and learning. Several studies have addressed the issue of students’ perceptions and misconceptions regarding climate change. Some studies have used large samples to explore the drivers of pro-environmental behaviour^88^, but small-group, classroom-based, or program-based studies are also frequent approaches to explore the different ways how students understand climate change^89–91^. As mentioned below, defining, and clarifying the required capabilities and the necessary skills to accelerate the implementation of the SDGs in higher education is essential^92^. A growing number of studies have addressed this issue, using different strategies such as comprehensive approaches based on literature review techniques and the use of surveys^87^, as well as small-group studies relying on quantitative and qualitative techniques^93^. In addition to this, non-conventional, student-centered teaching and learning techniques are becoming increasingly popular in higher education due to their potential to help students acquire multiple learning outcomes^94^. Methodologies such as problem-based learning^95–97^, inquiry-based learning^98^, gamification^99,100^, or participatory case studies^101^, are examples of innovative strategies to promote education for sustainable development.

**Table 3:** Case studies on teaching and learning.

Example	Purpose	University	Country	References
﻿Teaching resources and materials to visualize the chemistry of climate change	Model how rich frameworks can be applied to enable learning of general chemistry	Several	Canada and the United States	^90^
Workshop on the evaluation of the SDGs as a political action plan	Study of the SDGs as a political action plan	University of Kiel	Germany	^95^
Classroom-based intervention-oriented evaluation	﻿Identify and correct students’ misconstructions of climate change	Nanyang Technological University	Singapore	^89^
Adapted Delphi study	To develop key competence indicators for a course on ‘environment’	Seoul National University and National Youth Policy Institute	South Korea	^93^
The survey applied with university students	Understand the influence of psychological factors on pro-environmental behaviour	Several	Colombia and Nicaragua	^88^
Global online survey	﻿Explore degree of use of the SDGs by higher education institutions	Several	Global	^86^
Use of gamification strategies ﻿to simulate three spheres of economic interaction	﻿Rise graduates’ awareness about social and environmental outcomes of economic decisions	University of Salamanca	Spain	^130^
Collaborative transdisciplinary case study with students from the global North and global South	Exploring a prototype of a sustainability learning lab	University of Seychelles (UniSey) and ETH Zurich, Switzerland	Seychelles and Switzerland	^101^
Case study on student perceptions of climate change	Examining the views of university students of Small Islands Developing States	University of the South Pacific	Fiji	^91^
Case study on teaching climate change across schools and disciplines at a public university	Exploring interdisciplinary lecturer collaboration on teaching climate change	University of Tasmania	Australia	^98^

### Governance, operations, and institutional practice

All around the world, universities are increasingly adopting carbon–neutral goals and practices^102,103^. This is reflected by the growing number of higher education providers that are aiming to become fully carbon–neutral institutions (through low-carbon operational practices) while at the same time innovating their curricula to better educate students (about the benefits of carbon neutrality)^1,12,104^. With this twin strategy, universities are decreasing their own “carbon footprint” (by lowering institution-linked greenhouse gasses) and increasing the wider community’s “carbon brain print” (by teaching about low-carbon living)^102,105^. The literature, therefore, categorises “governance” into matters about the immediate institutional governance and operational practices (of the universities themselves) and their secondary flow-on function of informing and influencing the governance and operational practices of other key stakeholders beyond their organisational confines (e.g., local communities, national governments, and the corporate sector)^106^. Table 3 shows a set of selected case studies addressing areas of governance, operations, and institutional practice. In terms of facilitating institution-wide carbon neutrality, universities are implementing a raft of strategies that may include private-private solar system partnerships^102^, renewables, electric vehicles, tree plantation and enhanced energy efficiency^107^, remote sensing, and campus tree surveys to maximise biosequestration and campus-based ecosystem services^108^, campus community gardening to enhance CSR and institutional sustainability practice^109^, in addition to a range of other priority actions that may achieve net zero carbon buildings and (Paris-aligned) carbon reduction targets^102,110^. Furthermore, many universities have announced institutional commitments to divest their endowments from fossil fuel holdings while recalibrating their operational practices in alignment with the UN SDGs^111–113^. These actions may have image-enhancing effects^114^. Pertinent performance metrics are captured by the Times Higher Education (THE) Impact Rankings, an annual process that assesses universities against the UN SDGs. In its most recent fourth edition, THE has ranked a total of 1406 universities from 106 countries/regions^115^. Finally, additional information on the strategies, operations, and budgetary plans of higher education institutions (HEIs) regarding their transition to net zero or carbon neutrality might not be found in academic publications but could be available in “grey literature” produced by the HEIs themselves, focusing on their performance and strategic vision. Table 4 includes a set of selected case studies on governance, operations, and institutional practice.

**Table 4:** Case studies on governance, operations, and institutional practice.

Example	Purpose	University	Country	References
The Inter-University Sustainable Development Research Programme	Promotion of climate change and sustainability research around the world	Manchester Metropolitan University	United Kingdom	^87^
Advancing institution-wide carbon neutrality at an independent higher education provider	Implementation of private-private solar system partnership via power purchase agreement (PPA)	Christian Heritage College	Australia	^131^
Multipronged approaches to achieving institutional carbon neutrality	Attaining carbon neutrality via carbon reduction, offsetting, and teaching and research	Ernst-Moritz-Arndt-Universität Greifswald	Germany	^12^
Institutional strategies and operational practices to implement carbon neutrality	Energy consumption between 2012 and 2020 to assess its progress	University of Exeter	United Kingdom	^103^
Operational practices targeting and progressing institutional carbon neutrality	Implementing priority actions to achieve net zero carbon buildings and carbon reduction targets	University of New South Wales	Australia	^102^
Multipronged strategies to enhance operational practices and foster institutional carbon neutrality	Calculation of carbon storage, bio-sequestration, and values of campus-based ecosystem services	University of Michigan	United States	^108^
Carbon neutral and sustainable campus	Identifying mitigation strategies to maximise campus sustainability	NED University of Engineering and Technology	Pakistan	^107^
Influencing university governance toward sustainability	Implementing a more sustainable campus through divesting endowments from fossil fuel holdings	Massachusetts Institute of Technology	United States	^113^
Engaging students and staff in climate action	Implementing collective action on UBC’s Climate Action Plan	University of British Columbia	Canada	^110^
Enhance institutional sustainability practice	Documented experiences to promote social responsibility (CSR) and sustainability	Christian Heritage College	Australia	^109^

### Civic engagement and community outreach

The crucial role that Universities must play is also reflected by the increasing efforts from higher education institutions to foster civic engagement and expand their community outreach. Universities play a vital role in fostering civic commitment and community outreach within their localities. By leveraging their resources, expertise, and diverse talent pool, universities can initiate impactful initiatives that address community needs and promote positive social change. One effective approach is to establish university-community partnerships, where faculty, students, and staff collaborate with local organizations and residents to identify pressing issues and co-create sustainable solutions. Additionally, universities can integrate service-learning programs into their curricula, encouraging students to actively engage with the community while applying their academic knowledge to real-world challenges. Offering workshops, seminars, and public events on relevant topics further encourages dialogue and knowledge-sharing between the institution and the community. By actively involving themselves in the community's fabric, universities can contribute to the betterment of society, nurture socially responsible citizens, and empower students to become agents of positive transformation.

In this context, cooperation among stakeholders led to the concept of “co-creation for sustainability”, which deals with relevant notions and innovative strategies for transformative research^116^, such as participatory action research (PAR) and other community-based research strategies, the creation of urban living labs, and the use of innovative strategies for civic cooperation such as student service learning^117^. Table 5 shows a set of selected successful case studies where cooperation between universities and local stakeholders proved to contribute to facilitating civic engagement/community outreach in their local communities. Against this background, PAR, a community-based technique in which beneficiaries take an active role in research^118^, could be used for multiple purposes to deal with the challenges generated by climate change, such as improving climate planning processes^119^, increase engagement of different stakeholders and identify the scope for developing the adaptive capability of local communities^120^, monitor environmental risks and damage^121^, or strengthening climate justice^122^. Other forms of transformative research rely on multidisciplinary teams formed by diverse stakeholders engaged in evaluating knowledge and providing technical advice^123^ or fostering private–public partnerships to boost engineering solutions^124^, among other examples. In line with this multi-stakeholder cooperation, universities and other higher education institutions play a crucial role in creating ‘urban living labs’, understood as spaces where research is used for promoting innovation and collaboration to tackle social, economic, and environmental needs^125–127^. Finally, experiential learning strategies such as student service learning are becoming increasingly popular in higher education^128^, mainly because of their potential to foster critical thinking as well as promote social and civic engagement among students and enhance cooperation between different social actors^125, 129^.

**Table 5:** Case studies on civic engagement and community outreach.

Example	Purpose	University	Country	References
Urban labs as spaces for ﻿experimenting with new strategies to sustainability	﻿Explore the relevance on data collecting and place-based innovation for knowledge generation and local governance	Univ. of Manchester	United Kingdom	^126^
﻿Literature review on the history and progress of living labs	To evaluate to what extent living labs are useful to generate sustainable results	Deakin Univ	Australia	^127^
﻿Independent Advisory Committee	Discuss with actors to assess knowledge on climate action	Several	United States	^123^
﻿Using real data obtained from photovoltaic panels and the bus operation	﻿Minimize the operational costs of a sustainable charging station for an electric bus	Univ. of Campinas	Brazil	^124^
﻿Use of the campus open green space to boost knowledge acquisition	﻿Awareness about the efficacy of tree-planting projects to counterbalance carbon emissions	National Univ. of Singapore	Singapore	^125^
Identifying stresses confronted by farmers	﻿Supporting self-organization and co-learning processes	Univ. of Zimbabwe	Ghana and Zimbabwe	^120^
﻿Two PAR projects implemented by UN-Habitat and research associates	﻿Show the role of endogenous modes of resilience, and the correlation between them and sub-city climate vulnerability	Univ. of Melbourne, RMIT Univ	Australia, Solomon Islands, Vanuatu	^119^
A community-based project based on science land camps, capacity-building activities, and scientific data gathering	An environmental monitoring program to gather baseline data and foster local capacity-building	Univ. du Québec and Centre d'Études Nordiques	Canada	^121^
Multi-country study on institutional capabilities to support sustainability and climate justice	Foster institutional capabilities, influence governance, and inspire local actions	Univ. of South Pacific, Kenyatta Univ., Univ. of Passo Fundo	Brazil, Fiji and Kenya	^122^
Quantification of impacts of an environmental service-learning program	Measuring children’s attitudes and values toward the environment	Skidmore College, Univ. of Arizona, Univ. of Bayreuth	Mexico	^129^

## Conclusions

As this paper has outlined, universities can provide substantial contributions to both consumption and emissions globally. They also have the potential to play a key role in efforts to drive sustainability, both locally and globally.

As this paper has shown, many universities are switching to green operations that involve sustainability in campus activities such as water and energy consumption, waste production, and personal and institutional mobility, all of which have connections with climate change. Improvements may be pursued in respect of the implementation of activities such as smart waste management, sustainable transportation systems, and the more sustainable maintenance of existing buildings. Since daily campus operations result in the usage of large amounts of energy, it is important that higher education institutions that currently use fossil-fuel-based energy -which results in greater greenhouse gas releases- switch towards renewable energy use.

The production of waste also contributes greatly to global carbon emissions, and universities produce a significant amount of waste. Therefore, it is important to put in place appropriate strategies to manage waste and ideally prevent it, especially food waste since a large percentage of food wastage is generated at university canteens. There are ample examples of successfully-run recycling programmes, which may mobilise staff and students in a meaningful way. Some may not only reuse waste but also produce energy from it. Moreover, a further promising area is the use of cleaner transportation methods, as a tool to reduce the carbon footprint of higher education institutions. This may involve the use of campuswide shuttle services, carpooling, or the use of bicycles, by both staff and students. There is also much scope to reduce greenhouse emissions from travel. Whereas this is an essential part of universities´ operations -since both staff and students regularly use travel as part of their mobility and to attend conferences. Here, adequate solutions are also needed, for instance, the optimisation of trips and routes, and greater use of online facilities for those events whose physical attendance is not essential.

Higher education institutions can take several steps to address climate change. Such steps range from curriculum reform to creating new research initiatives and collaborations. Some of the recommendations that may further the cause of a greater engagement of universities on climate change include:

1. Curriculum Reform: as it is shown in Table 3, there are few studies focusing on climate change aspects in curriculums, indicating a large opportunity for research. In this sense, higher education institutions should review their curricula to ensure that current and future generations of students are educated in the fundamentals of climate science (in technical subjects) and the global effects of climate change (in non-technical ones).
2. Education & Awareness: Aligned with the first recommendation, institutions should promote educational campaigns and public awareness initiatives to educate students and the public on the importance of reducing their carbon footprint. The case studies on civic engagement and community outreach shown in Table 5 evidence interesting examples of ways of implementing this kind of initiative for the public in general. As presented in the literature, the complexity and inter-transdisciplinary character of sustainability inhibits its understanding to some extent, but concrete examples of carbon footprint reduction can be an important approach to address this challenge.
3. Research: There are relevant challenges highlighted in the literature for inserting climate change in university programmes, evidencing the need for studies to deeply analyse these difficulties and propose manners for overcoming them. In addition, the existing research institutions, initiatives, and/or programs focusing on climate change-related aspects (Tables 1 and 4) can play a key role in enhancing the efforts in the field and institutions worldwide should encourage and fund research initiatives that seek to understand the causes and effects of climate change, develop solutions and technologies, and identify innovative strategies for addressing the climate crisis.
4. Collaboration: In the same line of reasoning of the previous recommendation, institutions should establish and enhance partnerships with local governments, non-profit organizations, and other stakeholders to collaborate on initiatives to mitigate climate change.
5. Renewable Energy: Another evidenced source of improvement opportunity regarding climate change is that institutions should invest in renewable energy sources, such as solar, wind, and geothermal, to reduce their emissions and promote sustainability. They should, in other words, practice what they preach.
6. Green Buildings: Aligned with the previous recommendation, institutions should strive to create and maintain sustainable buildings, such as LEED-certified buildings, to reduce their environmental impact. In Table 4, examples of case studies on governance, operations, and institutional practice are evidenced, which can be used as a starting point for further development.

This paper has some limitations. The first is related to the sample. The study analysed 1214 documents that only considered how climate change education and/or research is pursued by universities, without focusing on other parameters. Secondly, only 794 documents remained in the database and were used for term co-occurrence analysis in VOSviewer. In addition, the case studies focused on four areas, namely research and development, teaching and learning, governance and operations, and civic engagement and community outreach, and did not consider elements such as collaboration with external organisations. Finally, the authors used the main terms to create the search string, and because of the diversity of the field, it was not possible to track all the possible terms related to each one of the four dimensions. However, this last limitation could be an opportunity for future studies as new terms that are not commonly used till the date of this research might start to gain attention and start to be adopted. Despite these limitations, the paper provides a welcome addition to the literature since it documents and promotes the current emphasis given by universities to climate change.

As to future trends on climate change and universities, there is a perceived need for greater engagement. Universities are important hubs of innovation and knowledge creation, with a comprehensive body of information and experience, which can significantly help to address the challenges of climate change, and across several subjects and contexts. As such, more universities are expected to intensify their efforts in research, education, and outreach activities related to climate change.

Moreover, universities should become more involved in the public policy and advocacy sphere, advocating for solutions to climate change and engaging in climate-related projects. This involvement is expected to increase as universities become more involved in the global climate change discourse.

### Supplementary Information

Supplementary Information.

## Data Availability

All data generated or analysed during this study are included in this published article [and its supplementary information files].
