# Supplementary material for: Towards a greater engagement of universities in addressing climate change challenges
Source: Sci Rep. 2023 Nov 3;13:19030. doi: 10.1038/s41598-023-45866-x (PMC10624841; doi:10.1038/s41598-023-45866-x)
Supplement: Supplementary file 1 — Supplementary Information. [file 41598_2023_45866_MOESM1_ESM.docx]

# Appendix

Search string used to perform the literature review

TS= (("clim* change" OR "global warming" OR "climate variability") AND ("extreme event" OR "extreme weather" OR "heat wave" OR “heat stress” OR "sea level*" OR “flood*” OR “drought*” OR “storm*” OR “hurricane*” OR “hotter temperature*” OR “Increased temperature*” OR “rising ocean*” OR “ocean acidification” OR “loss of species” OR “biodiversity” OR “desertif*” OR “Precipitation” OR “food” OR “health” OR “water” OR “wildfire” OR “migration” OR “displacement” OR “migrant*” OR “poverty” OR “conflict*” OR “ecosystem*” OR “fisher*” OR “forest*” OR “infrastructure”) AND (“educati*” OR “training” OR “teaching” OR “curricula” OR “curriculum” OR “program*” OR “research”) AND (“universit*” OR “higher education institut*” OR “college*” OR “academic institut*” OR “academia”))
